# Large-scale Quality Control of Cardiac Imaging in Population Studies: Application to UK Biobank

**DOI:** 10.1038/s41598-020-58212-2

**Published:** 2020-02-12

**Authors:** Giacomo Tarroni, Wenjia Bai, Ozan Oktay, Andreas Schuh, Hideaki Suzuki, Ben Glocker, Paul M. Matthews, Daniel Rueckert

**Affiliations:** 1 Imperial College London, Department of Computing, BioMedIA Group, London, SW7 2AZ UK; 2 City, University of London, Department of Computer Science, CitAI Research Centre, London, EC1V 0HB UK; 3 Imperial College London, Department of Brain Sciences, London, W12 0NN UK; 4 UK Dementia Research Institute, London, W12 0NN UK

**Keywords:** Interventional cardiology, Computer science

## Abstract

In large population studies such as the UK Biobank (UKBB), quality control of the acquired images by visual assessment is unfeasible. In this paper, we apply a recently developed fully-automated quality control pipeline for cardiac MR (CMR) images to the first 19,265 short-axis (SA) cine stacks from the UKBB. We present the results for the three estimated quality metrics (heart coverage, inter-slice motion and image contrast in the cardiac region) as well as their potential associations with factors including acquisition details and subject-related phenotypes. Up to 14.2% of the analysed SA stacks had sub-optimal coverage (i.e. missing basal and/or apical slices), however most of them were limited to the first year of acquisition. Up to 16% of the stacks were affected by noticeable inter-slice motion (i.e. average inter-slice misalignment greater than 3.4 mm). Inter-slice motion was positively correlated with weight and body surface area. Only 2.1% of the stacks had an average end-diastolic cardiac image contrast below 30% of the dynamic range. These findings will be highly valuable for both the scientists involved in UKBB CMR acquisition and for the ones who use the dataset for research purposes.

## Introduction

The UK Biobank (UKBB) is a population-based prospective study established to allow detailed investigations of the genetic and environmental determinants of the diseases of middle and old age^1^. Its cohort consists of 500,000 voluntary participants, with ages ranging between 40 and 69 years, that were recruited between 2006 and 2010 across the UK. The baseline assessment included collection of blood, urine and saliva samples (allowing genetic phenotyping), physical and functional measurements and answers to a questionnaire on health and lifestyle. Follow-up will be then conducted both through repetition of the baseline assessment on a cohort subset and through linkages to routinely available national datasets. This wealth of data will foster the discovery and the understanding of unknown underlying links between clinical conditions and lifestyle, environmental and genomic factors across the population of the UK. Starting from 2014, 100,000 volunteers from the whole cohort were also enrolled for multi-modal imaging, including MR of the brain, the heart and the full body^1^. Acquisitions are performed in a multi-centre setting using standardised protocols. As far as cardiac MR (CMR) is concerned, the acquisition protocol includes long- and short-axis cine, aortic distensibility cine, tagging, coronal left ventricular outflow tract (LVOT) cine, aortic valve flow phase contrast sequence and T1 mapping^2^. At the time of the present study the acquisition is ongoing, with more than 20,000 participants already scanned. For its size, the consistency in the acquisition details and the amount of accompanying data, the CMR dataset from the UKBB has already become a reference dataset, adopted in many research studies with both methodological^3,4^ and clinical^5–7^ focuses, and this trend is likely to increase in the future.

**Figure 1:**
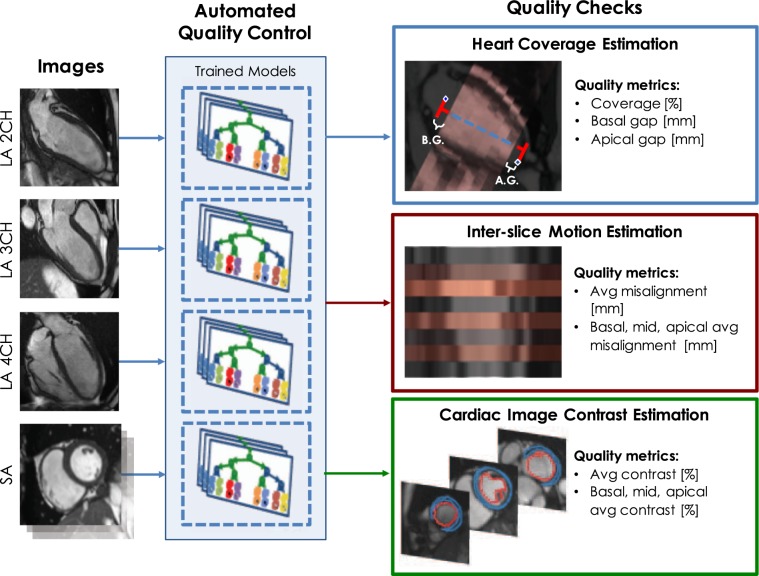
Overview of the automated quality control pipeline^19^. The pipeline estimates for each SA stack (1) heart coverage, (2) inter-slice motion, (3) cardiac image contrast. Coverage is defined as the percentage of the LV long axis which is covered by the SA stack; in addition, the potential gaps between the stack and the anatomical landmarks (i.e. mitral valve and apex for basal and apical regions, respectively) are also estimated. Inter-slice motion is defined as the average in-plane misalignment of the slices; the same quantity is also estimated separately for the basal, mid-ventricular and apical regions. Cardiac image contrast is defined as the average percentage of the dynamic range used in the slices to represent the difference in intensity between LV cavity and LV myocardium; regional quantities are similarly also estimated.

The quality of a CMR scan depends on the ability of the operator to correctly select the acquisition parameters (mainly relative to slice planning) in relation to the subject being scanned^8^ as well as on the occurrence of potential imaging artefacts (caused for instance by respiratory and cardiac motion, blood flow and magnetic field inhomogeneities)^9^. As a consequence, a quality control step is required to guarantee the usability of the acquired images. In clinical practice, this step is directly performed through visual inspection by the operator right after the acquisition. Besides being strongly subjective, visual quality control is a highly time-consuming task, and thus it does not fit into high-throughput acquisition protocols like the one of the UKBB. At the same time, the identification of sub-optimal or unusable scans is necessary to ensure the reliability of the results of subsequent analyses performed on the dataset. Accordingly, there is strong need for techniques for automated quality assessment of CMR images. Previous research efforts towards automated quality assessment of MR images have mostly focused on the estimation of noise levels^10,11^. However, most aspects relative to the quality of a scan are inherently modality-specific. Regarding CMR, very few attempts have been made towards the development of comprehensive quality control pipelines^12^. Most recent research studies have focused on automated heart coverage estimation for cine short-axis stacks, in order to identify whether the slices in a given stack cover the whole left ventricle (LV) or not^13–15^. Of note, these studies have been developed and tested on the UKBB, further demonstrating the importance of this assessment. Another highly investigated issue is respiratory motion^16^, whose main impact on cine stacks is in the form of inter-slice misalignments caused by differences in the breath-holding positions maintained during image acquisition^17^. Finally, another study on quality control for the CMR scans has been presented and tested on the UKBB^4^, but it focused on quality assessment of the segmentations obtained with an automated method rather than of the images themselves. An automated pipeline for image processing and quality control of the brain scans of the UKBB has been recently presented^18^: the quality control portion uses handcrafted features (e.g. volume, symmetry and intensity distributions of automatically segmented brain structures) to identify problematic scans. The authors applied the pipeline to 10,098 brain scans of the UKBB, and reported issues in 174 of them. However, their quality control technique targets the output of their image processing pipeline (which includes for instance image registration steps), and thus the reported metrics cannot be used to directly infer the quality of the raw brain scans. To the best of our knowledge, no extensive quality control assessment has been carried out on the currently available UKBB CMR scans. We have recently developed a learning-based, fully-automated quality control pipeline for short-axis (SA) cine stacks (Fig. 1) that estimates (1) heart coverage (defined as the percentage of LV long-axis actually covered by the SA stack), (2) inter-slice motion (defined as the average in-plane misalignment in mm of the SA slices) and (3) image contrast in the cardiac region (defined as the percentage of the dynamic range used to represent the difference in intensity between LV cavity and LV myocardium)^19^. The technique is based on hybrid random forests and was validated on up to 3000 scans from the UKBB against manual annotations and visual inspections performed by experienced interpreters.

In this paper, we present the results of the application of our automated quality control pipeline^19^ to the first batch of nearly 20,000 CMR scans from the UKBB. The aims of the present study are essentially three: first, to assess the reliability of the UKBB CMR scans; second, to identify potential trends relative to changes in image quality over time for the UKBB; third, to identify potential correlations between image quality and other factors such as acquisition details and non-imaging phenotypes of the subjects, including lifestyle variables and previous clinical assessments. We believe the obtained results are highly informative both for the UKBB scientists involved in the ongoing acquisition process as well as for the researchers who will use the dataset in the future for their research projects.

## Results

**Figure 2:**
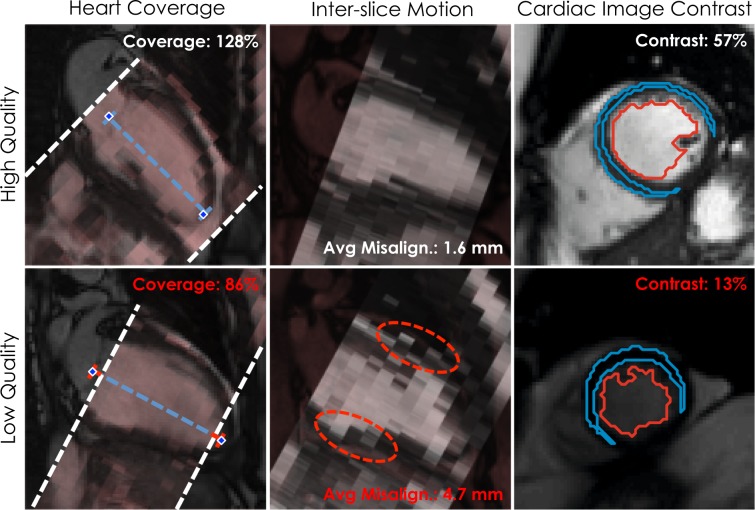
Examples of results obtained for the three quality checks in both high quality (top row) and low quality (bottom row) SA stacks. For the first two checks, the SA stacks (in red) are superimposed to the respective LA 2-chamber views for reference, while for the third one slice-based results are shown with the automatically extracted contours used to perform the estimation. For heart coverage, while in the top row both the landmarks (mitral valve and apex) are covered by the SA stack, in the bottom one they are both slightly outside, thus indicating a sub-optimal coverage. For inter-slice motion, in the top row the LV is well-aligned throughout the slices, whereas in the bottom one some slices are clearly misaligned (red dotted lines). For image cardiac image contrast, the top row exhibits well-defined contours, while the bottom one shows barely intelligible ones. Importantly, in all cases, differences in quality are well represented by the estimated metrics.

Out of the available 19,265 cases, the mentioned three quality control checks were performed on the 19,249 that contained all the expected images. The images missing from the 16 incomplete scans were, in total, 5 SA stacks, 11 LA 2-chamber views, 7 LA 3-chamber views and 8 LA 4-chamber views. The obtained results are here reported, while some examples are displayed in Fig. 2.

### Heart coverage estimation

**Figure 3:**
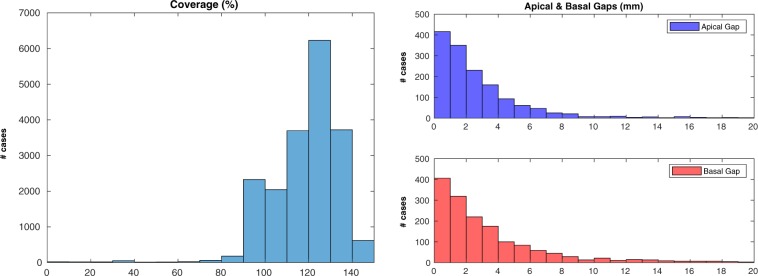
Coverage estimation - Overall results. Heart coverage (left) and apical/basal gaps (right) in the whole dataset. A non-negligible portion of the SA stacks has sub-optimal coverage (14.2% of the stacks are below 100% coverage).

**Figure 4:**
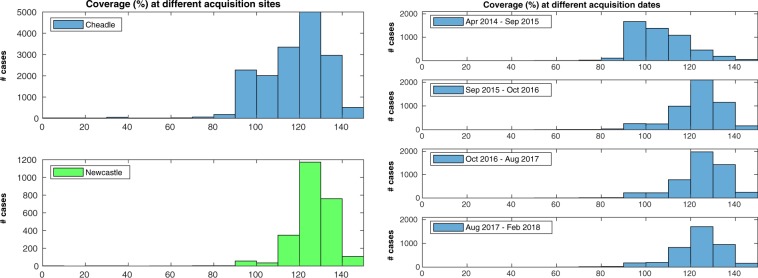
Coverage estimation - Association with acquisition details. Differences in heart coverage based on acquisition site (left) and acquisition date (right). Stacks acquired in Cheadle were apparently affected by more coverage issues than those acquired in Newcastle. Moreover, the scans acquired in the first year had substantially lower coverage than later ones.

The first check was performed on 19,129 cases: 120 cases (0.6% of the total) were excluded due to failing the sanity check implemented in the pipeline (please refer to the Methods for details). The results for heart coverage (Fig. 3, left) showed that while the majority of SA stacks were covering the whole LV or more (16412 cases, 85.8%, with coverage equal or greater than 100%), a non-negligible portion of them had sub-optimal coverage (2717 cases, 14.2%, below 100%; 390 cases, 2.0%, below 90%). The results for apical and basal gaps (Fig. 3, right) indicate that the two types of gaps were very similarly distributed (Wilcoxon rank sum test between apical and basal gaps: *p* = 0.0438, 95% confidence interval of the difference between medians *CI* = [−0.28, 0.00] mm): this suggests that they essentially occurred with equal probability and were of comparable entity. The associations between heart coverage and acquisition details such as site and date of acquisition were then assessed. Relatively to the acquisition site (Fig. 4, left, and Table 1), it appears that the stacks acquired at the UKBB facility located in Cheadle, UK, were more likely to be affected by coverage issues than those acquired in Newcastle, UK. The percentage of scans with coverage lower than 100% was 15.9% in Cheadle, but only 2.8% in Newcastle. Differences in acquisition date also were associated with differences in coverage (Fig. 4, right, and Table 1): the scans acquired in the first year of MR imaging had substantially lower coverage in comparison to the later ones. The correlation between heart coverage and physical measurements was also assessed: no correlation was found either with weight or with body surface area (BSA, see Table 1).

**Table 1:** Results of the statistical analyses for coverage.

	Coverage		
	p	95% CI (%)	τ_B_
Cheadle vs Newcastle	<10^−169^	[−7.5, −6.5]	
First year vs last year (*)	<10^−270^	[−18.4, −17.4]	
Weight (*kg*)	0.12		0.01
BSA (*m*^2^)	0.02		0.01

The rows with two contrasting groups show the results of a Wilcoxon rank sum test between them for coverage, whereas the rows with a single variable show the results of Kendall’s Tau-b rank correlation between that variable and coverage (more details can be found in the Statistical Analysis). (*): “first year” actually indicates the “Apr ‘14 – Sep ‘15” acquisition period, while “last year” indicates the “Aug ‘17 – Feb ‘18” period.

### Inter-slice motion estimation

**Figure 5:**
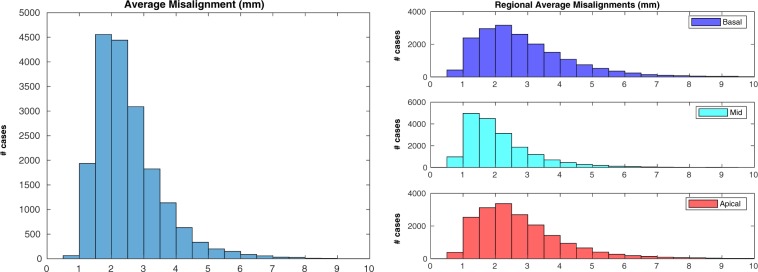
Motion estimation - Overall results. Average (left) and regional average (right) misalignments in the whole dataset. Apical and basal regions appeared slightly more affected by motion than the mid one (please refer to the Discussion for more insights on this aspect).

**Figure 6:**
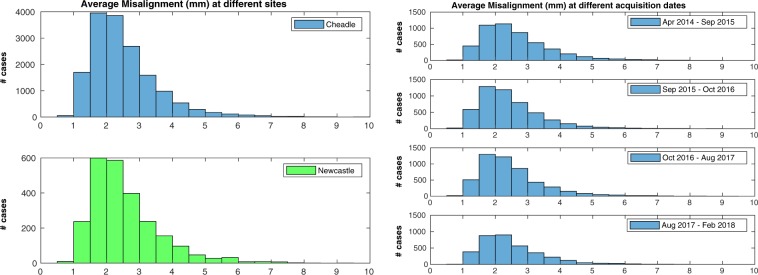
Motion estimation - Association with acquisition details. Differences in average misalignment based on acquisition site (left) and acquisition date (right). Neither site nor date seemed associated with relevant changes in average misalignment.

**Figure 7:**
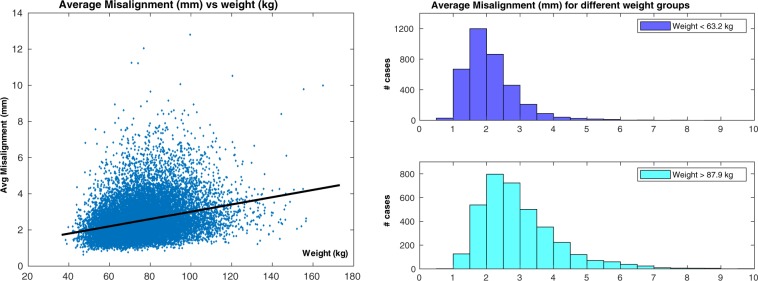
Motion estimation - Association with weight. Linear regression analysis between average misalignment and weight (left) and histograms representing average misalignment in the first and last quintile for weight, respectively (right). Weight was mildly correlated to average misalignment.

The second check was performed on 18,598 cases: 651 cases (3.4%) were excluded due to failing the sanity check implemented in the pipeline (please refer to the “Methods” for details). Inter-slice misalignment (Fig. 5, left) had a median value of 2.29 mm and an interquantile range (IQR) of 1.17 mm. The average regional misalignments (Fig. 5, right) suggest that the apical and basal regions were slightly more affected by motion than the mid one (average misalignment in the apical region: median = $2.48$ mm, IQR = $1.65$ mm; mid region: median = $1.86$ mm, IQR = $1.21$ mm; basal region: median = $2.56$ mm, IQR = $1.79$ mm. Results of the rank sum tests reported in Table 2). In terms of the associations between average misalignment and acquisition details (Fig. 6 and Table 2), it seems that neither acquisition site nor acquisition date were associated with relevant changes in average misalignment. The correlation between average misalignment and physical measurements as well as anagraphic data was also assessed (Table 2). Weight (Fig. 7, left) was found to be mildly correlated with average misalignment, and stacks acquired in subjects with lower weight were less likely to be affected by high misalignments (Fig. 7, right). Almost identical results were found for the correlation between average misalignment and BSA. No correlation was found with age or blood pressure. Among lifestyle variables (Table 2), neither physical activity nor alcohol intake frequency seem to be related to inter-slice motion. However, smoking habits (Supplementary Fig. S1) were associated with a very small increase in average misalignment. Finally, the associations between average misalignment and the presence of self-reported cardiovascular and respiratory diseases were evaluated (Supplementary Fig. S2 and Table S1). The conditions that seemed to have the strongest association with average misalignment were myocardial infarction, angina and chronic obstructive pulmonary disease (COPD).

**Table 2:** Results of the statistical analyses for average misalignment. The same description of Table 1 applies. (**): “low weight” indicates “weight <63.2 kg” (first quintile), while “high weight” indicates “weight >87.9 kg” (last quintile).

	Average Misalignment		
	p	95% CI (*mm*)	τ_B_
Mid vs apical	${\mathrm{ < 10}}^{-270}$	[−0.57, −0.53]	
Mid vs basal	${\mathrm{ < 10}}^{-270}$	[−0.64, −0.60]	
Cheadle vs Newcastle	0.08		
First year vs last year (*)	${\mathrm{ < 10}}^{-8}$	[−0.15, −0.07]	
Weight (*kg*)	${\mathrm{ < 10}}^{-270}$		$0.21$
Low weight vs high weight (**)	${\mathrm{ < 10}}^{-270}$	[−0.77, −0.69]	
BSA (*m*^2^)	${\mathrm{ < 10}}^{-270}$		$0.20$
Age	${\mathrm{ < 10}}^{-9}$		$0.03$
Systolic blood pressure (*mmHg*)	${\mathrm{ < 10}}^{-31}$		$0.06$
Diastolic blood pressure (*mmHg*)	${\mathrm{ < 10}}^{-39}$		$0.07$
Days/week with 10 + mins of walking	${\mathrm{ < 10}}^{-5}$		−0.03
Days/week with 10 + mins of vigorous physical act.	$0.13$		−0.01
Alcohol intake frequency	$0.0015$		−0.02
Never smoked vs currently smoking	${\mathrm{ < 10}}^{-10}$	[−0.28, −0.16]

### Cardiac image contrast estimation

**Figure 8:**
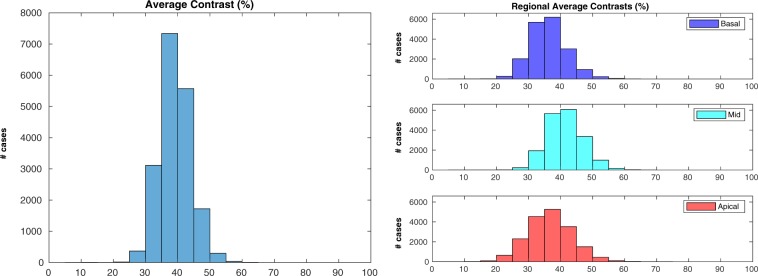
Contrast estimation - Overall results. Average (left) and regional average (right) contrasts in the whole dataset. Apical and basal regions appeared to have slightly lower contrast than the mid one (please refer to the Discussion for more insights on this aspect).

The third check was performed at end-diastole on 18,467 cases: 782 cases (4.1%) were excluded due to failing the sanity check implemented in the pipeline (please refer to the “Methods” for details). The results for average contrast (Fig. 8, left) were narrowly distributed around the median (median average contrast = $39$%, IQR = $6$%). Very few SA stacks had low average contrast (393 cases, 2.1%, with average contrast below 30%; 10 cases, 0.1%, below 20%). The regional assessments (Fig. 8, right) suggest that apical and basal slices had lower contrast in the cardiac region than mid ones (median average contrast in the apical region: median = $37$%, IQR = $9$%; mid region: median = $41$%, IQR = $7$%; basal region: median = $36$%, IQR = $7$%. Results of the rank sum tests reported in Table 3). Relatively to the association with acquisition site (Supplementary Fig. S3, left, and Table 3), it appears that the stacks acquired in Cheadle and those acquired in Newcastle had the same distribution for average contrast. Also acquisition date (Supplementary Fig. S3, right, and Table 3) seemed to have a negligible association with differences in contrast.

**Table 3:** Results of the statistical analyses for average contrast. The same description of Table 1 applies.

	Average Contrast	
	p	95% CI (%)
Mid vs apical	${\mathrm{ < 10}}^{-270}$	[5, 5]
Mid vs basal	${\mathrm{ < 10}}^{-270}$	[5, 5]
Cheadle vs Newcastle	0.65	
First year vs last year (*)	${\mathrm{ < 10}}^{-42}$	[1, 2]

## Discussion

The UKBB is a population-wide study with different acquisition facilities and a lifespan of more than a decade, dealing with both healthy subjects and prospective patients in a high-throughput setting: under these conditions, ensuring quality consistency of the acquired data is important yet extremely challenging. While the entire study has been designed keeping this goal in mind, perfect standardisation is clearly impossible to achieve. For what entails CMR imaging, several factors can have a negative impact on image quality. First of all, different operators can have different opinions on what constitutes an optimal scan, leading to different choices for the acquisition parameters (e.g. the number of acquired SA slices) and consequently to different images. In addition, subject preparation plays a crucial role in CMR imaging (especially in order to reduce bulk and respiratory motion artefacts), and some subjects can be less cooperative. Finally, there are some factors that are essentially out of the control of both the operator and the patient (like the presence of arrhythmias, which can lead to poor electrocardiogram triggering)^9^.

In this study, a series of experiments were performed to quantify image quality for the whole dataset and to assess potential associations between quality and factors relative to acquisition details and non-imaging phenotypes. While in an ideal dataset quality would be completely independent from these factors, some associations were identified.

### Heart coverage

Results show that 14.2% of the stacks did not achieve full coverage. Although this could seem very high, our results show that coverage issues were essentially limited to the first year of CMR imaging, when the acquisitions were all performed in the facility in Cheadle: in the following years, coverage values have substantially improved, and the recently introduced facility in Newcastle does not seem to be affected by the issue, potentially also thanks to the lower daily throughput requested during its initial phase. Of note, direct conversations with the imaging advisory board of UKBB have highlighted that the coverage issue in Cheadle was known and that steps had already been taken to solve it, which is confirmed by our results. No correlation was found between coverage and weight or BSA (despite the known correlation with heart size^20^), suggesting that the issues relative to coverage were essentially associated with the acquisition procedure and not to the characteristics of subject being scanned.

### Inter-slice motion

The results for motion estimation are less straightforward to interpret. To better assess the entity of the estimated average inter-slice misalignment, it is useful to keep in mind the in-plane spatial resolution for SA slices, which is 1.8 × 1.8 mm. Moreover, while validating our pipeline, an average misalignment of 3.4 mm or more was found to provide the highest accuracy in classifying stacks with “noticeable motion corruption”, identified as such by an experienced interpreter through visual assessment^19^. If this threshold is accepted, the number of motion corrupted stacks is 2977 (16.0%). This suggests that inter-slice misalignment is indeed an issue with relatively high incidence in the UKBB study. However, inter-slice motion does not have any negative effect on subsequent 2D analyses, limiting its impact. If analyses with 3D techniques are instead planned, it is important to remember that several motion correction techniques for CMR stacks have been developed in the past^7,21–23^ and could be applied to perform slice realignment in post-processing. At the same time, these techniques are able to perform only in-plane motion correction, while differences in breath-holding positions can cause complex roto-translations of the heart in all three dimensions^24^: as a consequence, caution must be exercised in performing motion compensation, and stacks with high average misalignment should be simply excluded from subsequent analyses.

As far as regional assessments are concerned, it seems that both the apical and basal region are statistically more affected by motion than the mid one, but this could be partially explained by the fact that the motion detection technique in these two regions is likely to be slightly less accurate and to overestimate the actual misalignment. Differently from coverage, acquisition site and date did not seem to be associated with variations in respiratory motion. On the other hand, weight and BSA were both positively correlated with average misalignment. This could be explained by the fact that people with higher BSA have bigger diaphragms^25^, and thus might be capable of producing bigger displacements of the heart with respiration^26^. Smoking habits were associated with an increased average misalignment, but the entity of the measured effects suggests that their impact is negligible.

Relatively to the associations with cardiovascular and respiratory pathologies, no strong correlations were identified. The condition with the strongest overall association with average misalignment seemed to be COPD, which can be explained with the typical symptoms associated with this pathology (e.g. shortness of breath). This suggests that additional care during patient preparation could be advisable when performing imaging on subjects affected by respiratory diseases to ensure the quality of the scan. Regarding this analysis, however, it is worth keeping in mind that the the presence of previously diagnosed conditions was provided in the UKBB by the subjects themselves through self-assessment (performed using an electronic questionnaire with the assistance of a trained nurse) and thus the obtained results in this regard should be interpreted with caution.

### Cardiac image contrast

The results relative to contrast estimation at end diastole seemed to be very positive. To decide on a minimum threshold for average contrast below which the usability of the scan would be compromised is a difficult task, highly dependent on the subsequent analyses: however, the obtained results suggest that contrast is generally acceptable in the vast majority of UKBB stacks. Regional assessments suggest that mid slices generally have higher contrast than basal and apical ones, however this also could be partially due to a slightly lower accuracy of the contrast estimation technique in these regions. Finally, factors such as acquisition site and date seem to have no relevant associations with variations in average contrast.

The present study is affected by some limitations. First, the adopted automated quality control pipeline is obviously subject to error: however, its previously reported validation on UKBB (e.g. sensitivity and specificity respectively 88% and 99% in the classification of stacks with coverage issues, 85% and 95% for stacks with motion corruption, Pearson’s correlation coefficient up to 0.95 for estimated contrast values vs reference measurements) indicate its high levels of accuracy^19^, thus making the considerations presented in this study likely to be realistic and reliable. Second, the pipeline is optimised for end-diastolic frames, and thus not applied to entire image sequences. While this is not a limitation for the first two quality checks (coverage needs to be assessed when the heart is fully dilated and inter-slice motion in breath-holding acquisitions is independent from the cardiac cycle), it limits the scope of contrast estimation. However, our pipeline specifically targets the overall appearance of the acquired sequences, and not transient image artefacts that can potentially appear during the cardiac cycle (e.g. in-slice motion corruption and flow artefacts), which should be assessed with different techniques and go beyond the scope of our analysis.

In summary, in this study we presented the results of the application of a recently developed automated quality control pipeline^19^ to the CMR images of the UKBB. Specifically, the pipeline is able to estimate heart coverage, inter-slice motion and cardiac image contrast for each SA cine stack. Potential correlations and associations between the estimated quality metrics and other factors (acquisition details and subject-related non-imaging phenotypes such as lifestyle variables and previous clinical assessments) have been evaluated: the results show that while quality metrics are generally high throughout the whole UKBB dataset, some small differences in quality were associated with a few factors (e.g. acquisition site and date for heart coverage, and weight and BSA of the subject being scanned for inter-slice motion). These results could be beneficial both to the scientists involved in data acquisition for large population studies like the UKBB as well as for those who use this valuable dataset for research purposes:

- Regarding acquisition, ensuring that coverage is properly addressed in the standard operating procedures across the different imaging centres, and that extra care is spent during patient preparation for overweight subjects, could contribute to maximise quality;
- Regarding the use of the dataset, it is advisable to double-check the scans acquired during the first year at Cheadle to identify stacks with sub-optimal coverage, and to perform inter-slice motion correction before running 3D analyses.

This paper was also a successful case study relative to the application of our automated quality control pipeline, which was proven useful in the off-line classification of sub-optimal scans and in the identification of suspected longitudinal trends in quality. Importantly, the pipeline could also be deployed at the acquisition site, allowing the on-line assessment of scan quality in the background: upon the detection of a sub-optimal scan, the operator could be alerted, allowing the modification of the acquisition settings (relatively for instance to acquisition parameters or patient preparation) and the triggering of a new acquisition. This would enable the improvement of the overall quality of the obtained scans, without the excessive costs demanded by visual assessment.

## Methods

### Data acquisition

The dataset used in this study consists of long- and short-axis cine CMR images of 19,265 subjects (61.7 ± 7.0 years, 52% female) extracted from the UKBB^1^. CMR imaging was performed using a 1.5 T Siemens MAGNETOM Aera system with a 18 channels anterior body surface coil (45 mT/m and 200 T/m/s gradient system). 2D cine balanced steady-state free precession (b-SSFP) short-axis (SA) image stacks were acquired with in-plane spatial resolution 1.8 × 1.8 mm, slice thickness 8 mm, slice gap 2 mm, image size 198 × 208 and average number of 10 slices. 3 standard 2D cine b-SSFP long-axis (LA) images (2-, 3- and 4-chamber views) were acquired for each subject with in-plane spatial resolution 1.8 × 1.8 mm, slice thickness 8 mm and image size 162 × 208. All of the reported details were consistent among the different UKBB acquisition sites (further details can be found in the literature^2^). Images were then converted from DICOM to NIFTI using the dcm2niix tool^27^.

To identify potential associations between image quality and other factors, additional data were downloaded for each subject from the relative data showcase (http://biobank.ctsu.ox.ac.uk/crystal/label.cgi). The selected variables are the following ones, reported with the UKBB field ID in brackets: sex (31), age (21003), date of imaging examination (53), site of imaging examination (54), weight (21002), body mass index (21001), body surface area (derived from weight and body mass index using the Mosteller formula), days/week with 10+ minutes of walking (864), days/week with 10+ minutes of vigorous physical activity (904), smoking status (20116), alcohol intake frequency (1558), systolic blood pressure (4080), diastolic blood pressure (4079), self-reported cardiovascular conditions diagnosed by a doctor (6150), non-cancer self-reported conditions (20002). Most of these variables have been collected multiple times throughout the acquisition of the UKBB: given the aim of the present study, we decided to use the values collected in conjunction with the follow-up visit for the imaging study (labelled as “2.0” in the relative spreadsheets) with the exception of the two variables relative to self-reported conditions (6150 and 20002), for which all of the multiple records were combined aiming for a more robust assessment. These two variables were used for the assessment of associations between average misalignment and presence of pathology. In particular, the “healthy” control group was created by using the first variable (6150) to select subjects who answered “none of the above” when asked about previously diagnosed infarction, angina, stroke or high blood pressure. The second variable (20002) was instead used to identify subjects that reported the following conditions: angina (illness code: 1074), infarction (1075), arrhythmia (1077), cardiomyopathy (1079), asthma (1111), chronic obstructive pulmonary disease (1112), emphysema (1113) and bronchiectasis (1114). These conditions were selected because of their potential impact on the cardiovascular and respiratory systems and thus on the capability of the subjects to successfully undergo breath-holding image acquisition.

### Automated quality control

Our automated quality control pipeline^19^ takes as input the SA stack and the three LA images acquired for each subject, and uses them to perform three quality checks: (1) heart coverage estimation, (2) inter-slice motion estimation and (3) cardiac image contrast estimation (Fig. 1).

Heart coverage estimation produces as output the coverage of the LV defined as the (percent) portion of the space between apex and mitral valve (i.e. the extrema of the LV, ideally marking the beginning and the end of the acquired volume) which is actually covered by the SA stack. Of note, coverage computation takes into account over-abundant stacks (which cover more space than the minimum required), producing a value greater than 100%. For cases of sub-optimal coverage, the quality check estimates also the distances in mm along the z-axis between the most basal slice and the mitral valve (referred to as “basal gap”) and/or between the most apical one and the LV apex (“apical gap”). The estimation is performed on the end-diastolic frame in order assess coverage when the heart is fully dilated. Finally, it is worth noting that the estimation is insensitive to potentially missing mid-ventricular slices: however, due to the specifics of the acquisition protocol of UKBB, this eventuality is extremely unlikely and was never encountered in our dataset.

Inter-slice motion estimation produces as output the average of the (absolute) in-plane slice misalignments in mm with respect to the reference position obtained from the three LA images. If the subject is able to perform subsequent breath-holds with his diaphragm always at the same position throughout the acquisition, then the shape of the LV in the slices of the SA stack will be realistic and consistent with the one in LA images. Otherwise, the LV will appear shifted in one or more slices: for each of them, the in-plane translation required to perform an approximate realignment is then estimated by the technique, and its magnitude in mm used as a measure of slice misalignment. The pipeline is able to restrict the assessment to the slices positioned between the apex and the mitral valve, and the final output is the average misalignment computed over them. In addition, regional assessments are also performed: apical, basal and mid average misalignments are estimated computing the average misalignment respectively of the first 2, last 2 and remaining slices of each stack. The estimation is performed on the end-diastolic frame without loss of generality, since inter-slice motion in breath-holding acquisitions is independent from the cardiac cycle. Finally, while strong respiratory motion can also have an out-of-plane component, it is of much lesser entity than the in-plane one^24^, which can thus be used as a proxy for global motion.

Cardiac image contrast estimation produces as output the (percent) difference at end diastole between the average intensity of the LV blood pool and that of the LV myocardium normalised by the dynamic range of the image, and then averaged over all the slices between the apex and the mitral valve. If only a small portion of the dynamic range is used to differentiate the blood pool from the myocardium, then the boundaries between these two structures are likely to be poorly defined, potentially hindering subsequent analyses. Similarly to motion estimation, regional assessments (apical, basal and mid average contrast) are also performed. This estimation is limited to the end-diastolic frame: the goal of this check is in fact to perform an assessment of the overall appearance of the sequence and not to identify potential transient image artefacts over the cardiac cycle.

These three checks are performed leveraging information that the quality control pipeline is able to extract from the SA stack and the LA images, specifically landmark positions for the mitral valve and the apex, and probabilistic segmentation maps for the LV blood pool and LV myocardium. Both landmark positions and probabilistic segmentation maps are extracted at once using hybrid decision forests, which extend the standard decision forest model to composite labels. Importantly, a series of sanity checks have been implemented to identify unrealistic or unreliable landmarks or segmentation maps and thus to exclude the relative scans from the automated quality assessment. For coverage estimation, the sanity check is considered failed if the landmarks from all three LA images are at unrealistic distances between each other. For motion and contrast estimation, the sanity checks are considered failed if the segmentation maps are deemed unreliable for the specific task or if less than 6 slices are estimated between the apex and the mitral valve. Of note, images that fail the sanity checks are likely to be affected by some sort of abnormality, and should thus be checked visually. For further details relative to the functioning of the pipeline (including training procedure, implementation of the sanity checks and limitations), please refer to the methodological paper^19^.

The learning-based portion of the pipeline was trained using 2 Intel Xeon CPU E5-2650 v2 @ 2.60 GHz with 220 GB of memory, which took approximately 5 days of computing time. The pipeline was then applied to the first 19,265 CMR scans of the UKBB, and the results of the three quality checks stored. Roughly 20 s were required to check one SA stack at a time (simulating an on-line application scenario) using an Intel Xeon CPU E5-1650 v3 @ 3.50 GHz with 64 GB of memory.

### Statistical analysis

Normality tests were performed independently for each of the three quality metrics using the Anderson-Darling test. Since the null hypothesis of normality was rejected for each of the three cases, non-parametric tests were selected for the statistical analyses, and median and interquantile range (IQR) were preferred to mean and standard deviation. A range of comparisons was performed to identify potential associations between quality metrics and variables extracted from the UKBB. The variables selected for these analyses were chosen heuristically based on their suspected influence on each specific quality metric (also as determined during conversations with the imaging advisory board of UKBB). The results of all the performed comparisons are either reported in the paper or in the supplementary material. To study the association between quality metrics and categorical variables (e.g. acquisition site), histograms and Wilcoxon rank sum tests (estimating also the 95% confidence intervals of the difference between medians) were used, while Kendall’s Tau-b rank correlation and linear regression analyses were performed to compare quality metrics with continuous variables (e.g. weight). For all of the mentioned statistical tests, the significance level was set to *α* = 0.05. Given the relatively small number of tests performed for both heart coverage estimation and cardiac image contrast estimation respectively, no correction for multiple comparisons was deemed necessary. On the other hand, the number of tests performed relatively to motion estimation were 19 (8 of which to assess associations with pathology): therefore, we corrected our analyses for multiple testing by means of the Bonferroni correction, yielding a threshold for significance *p*_*corr*_ = 0.0026.

### Ethics approval and consent to participate

UK Biobank has approval from the North West Research Ethics Committee (REC reference: 11/NW/0382). All methods were performed in accordance with the relevant guidelines and regulations, and informed consent was obtained from all participants. More information can be found on the UKBB resource catalogue page (http://biobank.ndph.ox.ac.uk/showcase/catalogs.cgi).

## Supplementary information

Supplementary Information.

## Data Availability

The images were provided by the UK Biobank Resource under Application Number 18545. Researchers can apply to use the UK Biobank data resource by submitting a proposal for health-related research in the public interest. Indicative fees inclusive of proposal submission and access to bulk data (including MR images) is £2250 + VAT (where applicable). More information can be found on the UKBB researchers page (https://www.ukbiobank.ac.uk/researchers/).
