# Supplementary material for: Large-scale Quality Control of Cardiac Imaging in Population Studies: Application to UK Biobank
Source: Sci Rep. 2020 Feb 12;10:2408. doi: 10.1038/s41598-020-58212-2 (PMC7015892; doi:10.1038/s41598-020-58212-2)
Supplement: Supplementary file 1 — Supplementary Information. [file 41598_2020_58212_MOESM1_ESM.pdf]

Large-scale Quality Control of Cardiac Imaging in Population Studies: Application to UK Biobank

Giacomo Tarroni[1,2*], Wenjia Bai[1], Ozan Oktay[1], Andreas Schuh[1], Hideaki Suzuki[3], Ben Glocker[1], Paul M. Matthews[3,4], and Daniel Rueckert[1]

[1]Imperial College London, Department of Computing, London, SW7 2AZ, U.K.
[2]City, University of London, Department of Computer Science, London, EC1V 0HB, U.K.
[3]Imperial College London, Department of Brain Sciences, London, W12 0NN, U.K.
[4]UK Dementia Research Institute, London, W12 0NN, U.K.
[*]First and corresponding author: giacomo.tarroni@gmail.com

ABSTRACT

In large population studies such as the UK Biobank (UKBB), quality control of the acquired images by visual assessment is unfeasible. In this paper, we apply a recently developed fully-automated quality control pipeline for cardiac MR (CMR) images to the first 19,265 short-axis (SA) cine stacks from the UKBB. We present the results for the three estimated quality metrics (heart coverage, inter-slice motion and image contrast in the cardiac region) as well as their potential associations with factors including acquisition details and subject-related phenotypes. Up to 14.2% of the analysed SA stacks had sub-optimal coverage (i.e. missing basal and/or apical slices), however most of them were limited to the first year of acquisition. Up to 16% of the stacks were affected by noticeable inter-slice motion (i.e. average inter-slice misalignment greater than 3.4 mm). Inter-slice motion was positively correlated with weight and body surface area. Only 2.1% of the stacks had an average end-diastolic cardiac image contrast below 30% of the dynamic range. These findings will be highly valuable for both the scientists involved in UKBB CMR acquisition and for the ones who use the dataset for research purposes.

Supplementary Information

	Average Misalignment		
	**# of cases**	**p**	**95% CI (*mm*)**
Angina	531	$< 10^{-8}$	$[0.15, 0.31]$
Infarction	387	$< 10^{-6}$	$[0.16, 0.34]$
Arrythmia	126	1	
Cardiomyopathy	12	0.04	$[0.02, 0.94]$
Asthma	2552	0.002	$[0.02, 0.09]$
COPD	138	$< 10^{-6}$	$[0.24, 0.54]$
Emphysema	233	0.0001	$[0.11, 0.34]$
Bronchiectasis	81	< 0.3	$[-0.08, 0.33]$

Table S1. Results of the rank sum tests between average misalignment in subjects with self-reported cardiovascular and respiratory diseases vs in the “healthy” control group (more details can be found in the Statistical Analysis in the main paper).

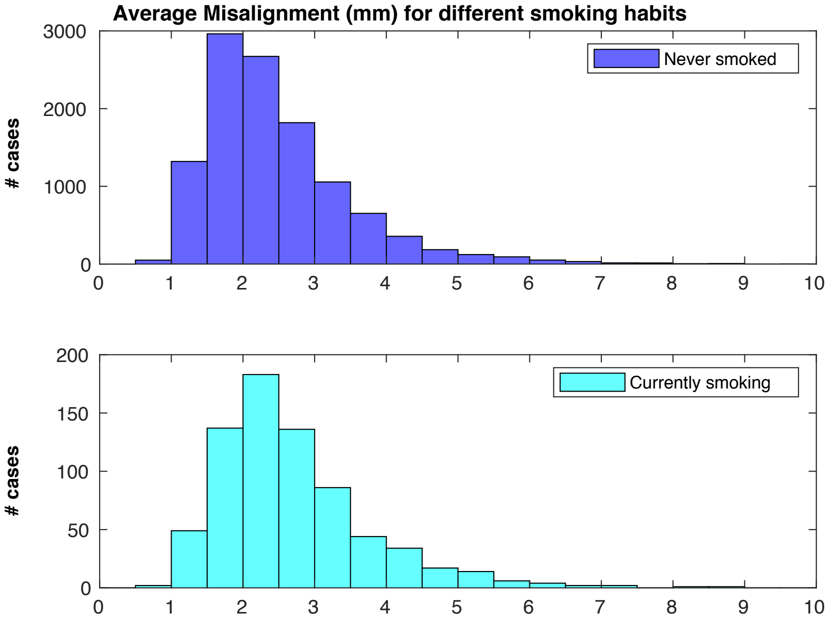

Figure S1. Motion estimation - Association with smoking habits. Histograms representing average misalignment in subjects who never smoked and who currently smoke, respectively. Smoking habits were associated with a small increase in average misalignment.

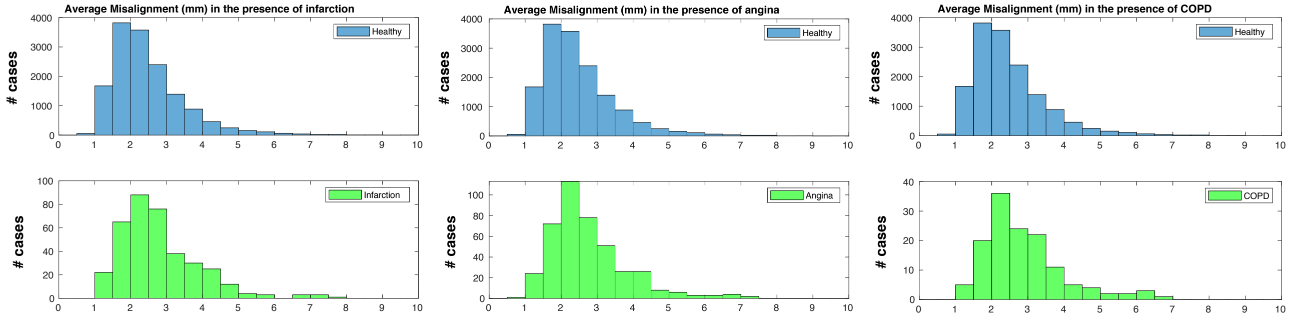

Figure S2. Motion estimation - Association with pathology. Histograms representing average misalignment in subjects with self-reported infarction (left), angina (middle) and COPD (right), respectively vs in the "healthy" group (more details can be found in the Statistical Analysis in the main paper). Among self-reported conditions, the three shown here seemed to have the strongest association with average misalignment.

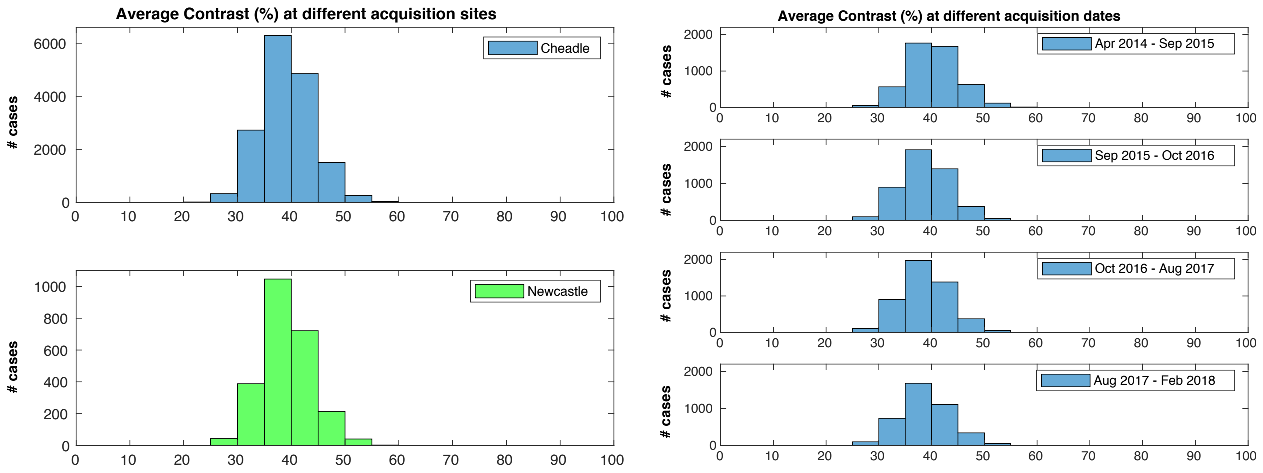

Figure S3. Contrast estimation - Dependence on acquisition details. Differences in average contrast based on acquisition site (left) and acquisition date (right). Neither site nor date seemed associated with relevant changes in average contrast.
